# Supplementary material for: Entry, replication and innate immunity evasion of BANAL-236, a SARS-CoV-2-related bat virus, in Rhinolophus and human cells
Source: PLoS Pathog. 2026 Apr 20;22(4):e1013573. doi: 10.1371/journal.ppat.1013573 (PMC13108884; doi:10.1371/journal.ppat.1013573)
Supplement: S1 Fig — (PDF) [file ppat.1013573.s001.pdf]

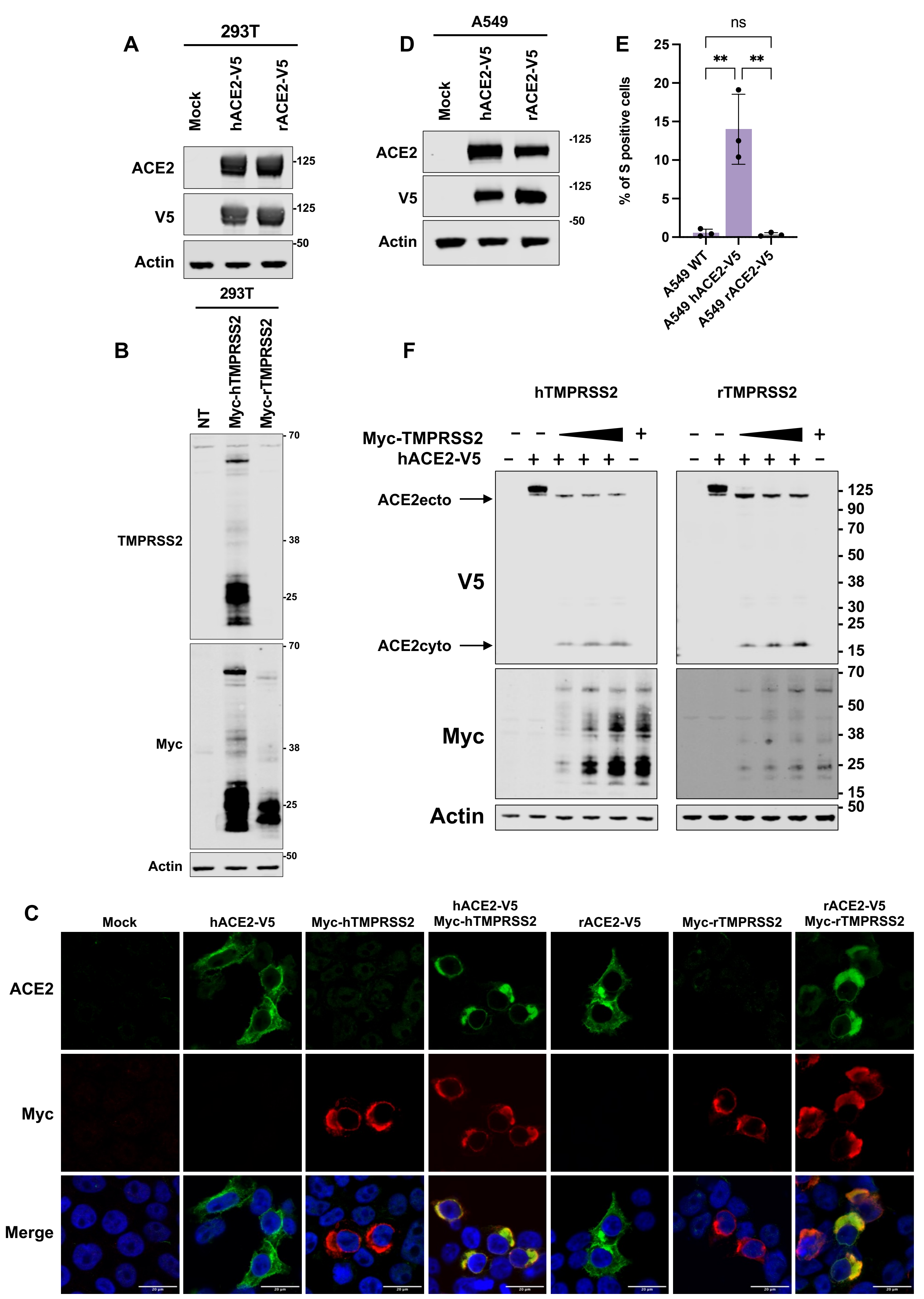

Figure S1. The tagged versions of ACE2 (ACE2-V5) and TMPRSS2 (Myc-TMPRSS2) are well expressed and functional. (A-B) 293T cells were transfected with plasmids expressing V5-tagged versions of hACE2 or rACE2 **(A)** or myc-tagged versions of hTMPRSS2 or rTMPRSS2 **(B)**. Whole-cell lysates were analyzed by western blotting 24 h post-transfection using antibodies against the indicated proteins. Data are representative of three independent experiments. **(C)** Confocal microscopy analysis of 293T cells transfected with ACE2-V5 (hACE2-V5/rACE2-V5) or/and myc-TMPRSS2 (myc-hTMPRSS2/myc-rTMPRSS2) expression plasmids. Cells were fixed and stained with ACE2 (green) and myc antibodies (red). Nuclei were stained with DAPI (blue). Data are representative of two independent experiments. Scale bar 20µm. **(D)** Western blot analysis of transduced A549 cells expressing V5 tagged hACE2 or rACE2 probed with antibodies against the indicated proteins. **(E)** A549 cells were infected with SARS-CoV-2 at a multiplicity of infection (MOI) of 1. Twenty-four hours later, cells were collected and the percentages of cells expressing the SARS-CoV-2 Spike protein (S) were determined by flow cytometry analysis. Data are means ± SD of three independent experiments. One-way ANOVA tests with Tukey's correction were performed. $^{**}P < 0.01$. **(F)** 293T cells were transfected with hACE2-V5 (0.5µg) and increasing amounts (0.25µg, 0.5µg or 1 µg) of myc-hTMPRSS2 (right panel) or myc-rTMPRSS2 (left panel). Western blot were probed with antibodies against the indicated proteins. Ecto, ectodomain; cyto, cytoplasmic domain. Data are representative of two independent experiments.
