# Supplementary material for: Entry, replication and innate immunity evasion of BANAL-236, a SARS-CoV-2-related bat virus, in Rhinolophus and human cells
Source: PLoS Pathog. 2026 Apr 20;22(4):e1013573. doi: 10.1371/journal.ppat.1013573 (PMC13108884; doi:10.1371/journal.ppat.1013573)
Supplement: S2 Fig — (PDF) [file ppat.1013573.s002.pdf]

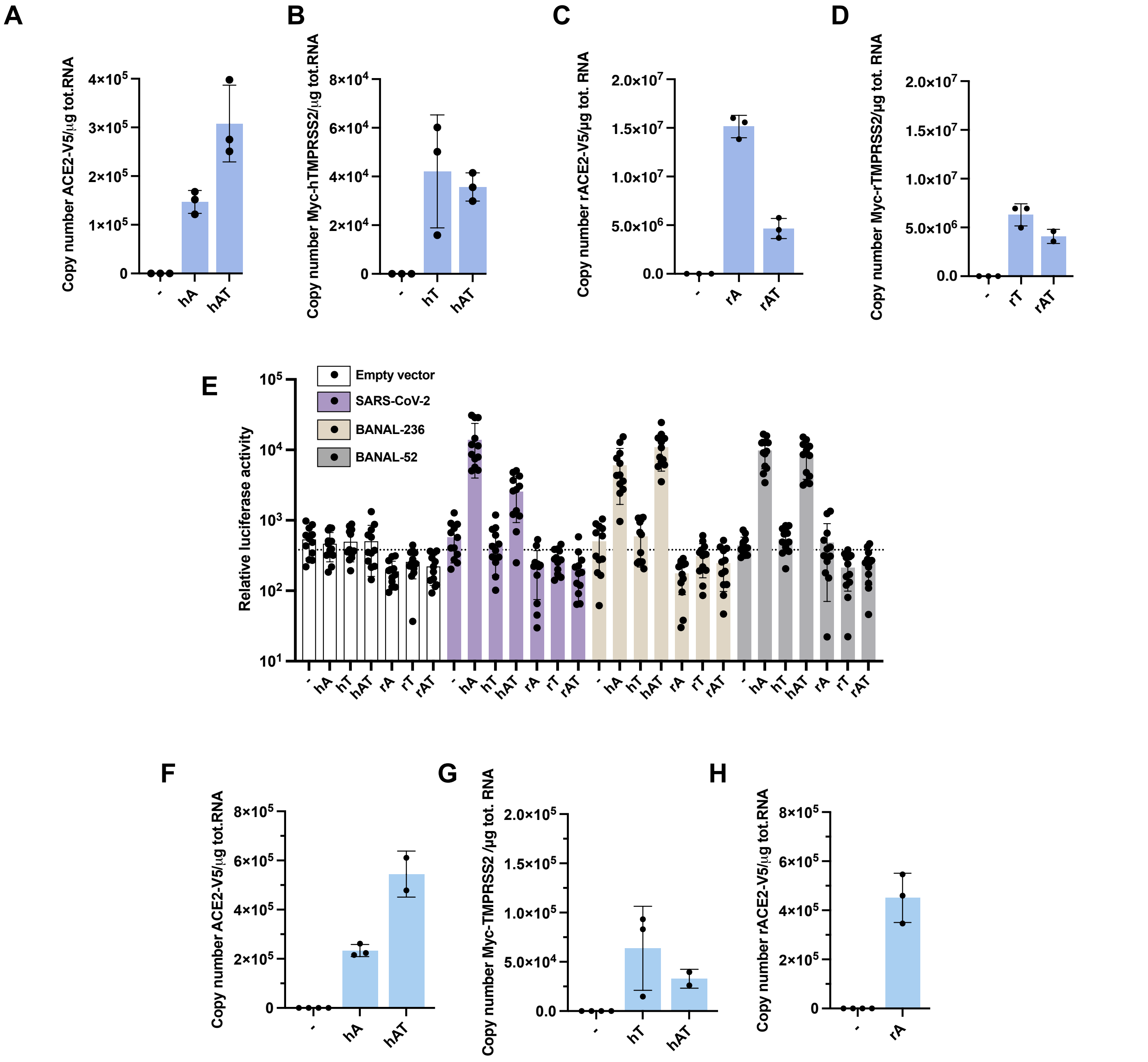

Figure S2. Investigations of BANAL-236 entry mechanisms in 293T, BHK-21 and RFe cells. (A-D) Quantification of copy numbers per µg of total cellular RNA of hACE2-V5 **(A)**, myc-hTMPRSS2 **(B)**, rACE2-V5 **(C)**, or myc-rTMPRSS **(D)** in 293T cells via qPCR analysis. Data are means ± SD of at least two independent experiments. **(E)** Pseudovirus entry assays were performed in wt BHK21 cells (-) or transiently expressing hACE2-V5 (hA), myc-hTMPRSS2 (hT), rACE2-V5 (rA), myc-rTMPRSS2 (rT), or both entry factors (hAT or rAT). Two days later, cells were transduced with the same HIV-1 p24 quantity of pseudo-lentiviruses bearing the S proteins of SARS-CoV-2 (purple), BANAL-236 (beige) or BANAL-52 (grey). Results are expressed in relative luminescence units (RLU). The dashed line indicates the average RLU obtained with cells transduced with empty vectors (white). Data are means ± SEM of four independent experiments. **(F-H)** Quantification of copy numbers per µg of total cellular RNA of hACE2-V5 **(F)**, myc-hTMPRSS2 **(G)** and rACE2-V5 **(H)** in Rfe cells via qPCR analysis. Data are means ± SD of at least two independent experiments.
