# Supplementary material for: Entry, replication and innate immunity evasion of BANAL-236, a SARS-CoV-2-related bat virus, in Rhinolophus and human cells
Source: PLoS Pathog. 2026 Apr 20;22(4):e1013573. doi: 10.1371/journal.ppat.1013573 (PMC13108884; doi:10.1371/journal.ppat.1013573)
Supplement: S3 Fig — (PDF) [file ppat.1013573.s003.pdf]

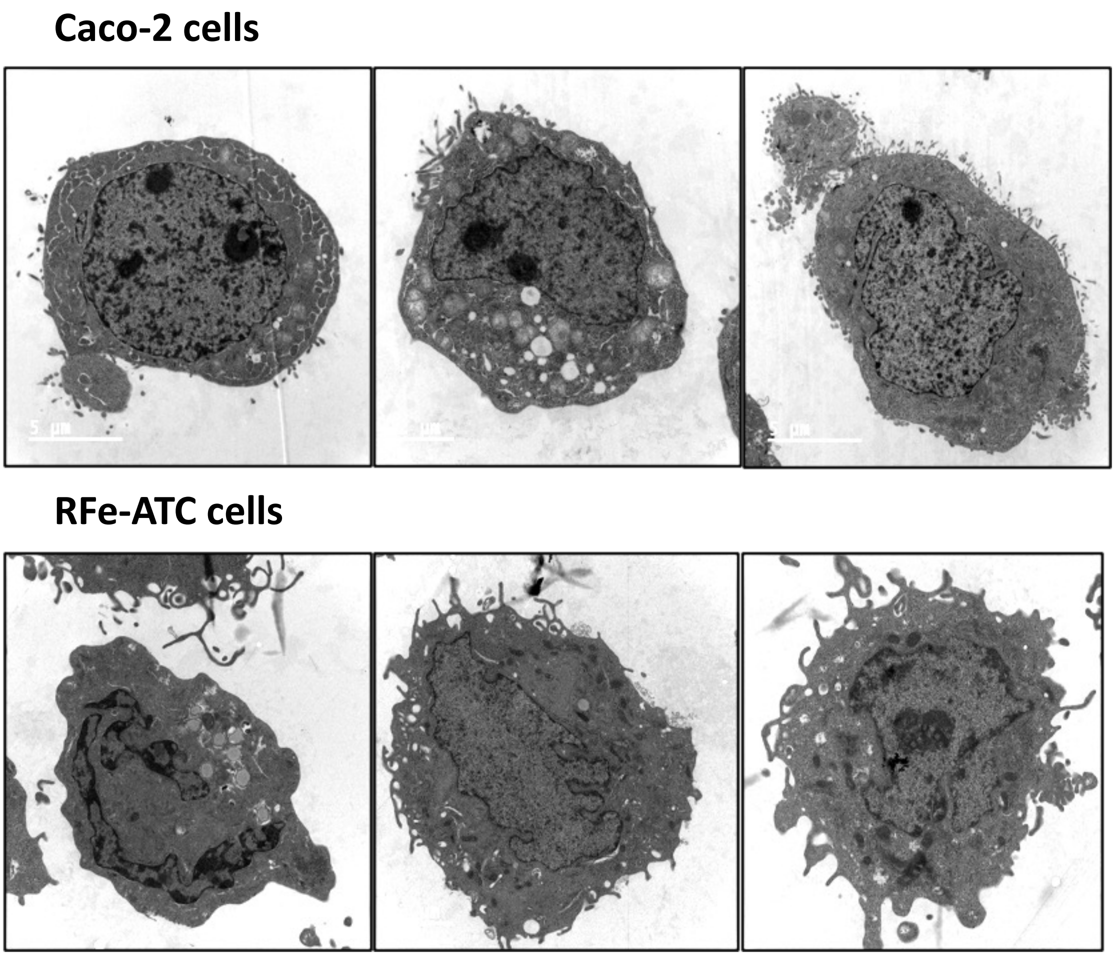

Figure S3. **Transmission electron microscopy analysis of Caco-2 and RFe-ATC cells**. Mock-infected cells were used as control cells for experiments shown in figure 5.
