# Supplementary material for: Entry, replication and innate immunity evasion of BANAL-236, a SARS-CoV-2-related bat virus, in Rhinolophus and human cells
Source: PLoS Pathog. 2026 Apr 20;22(4):e1013573. doi: 10.1371/journal.ppat.1013573 (PMC13108884; doi:10.1371/journal.ppat.1013573)
Supplement: S4 Fig — (PDF) [file ppat.1013573.s004.pdf]

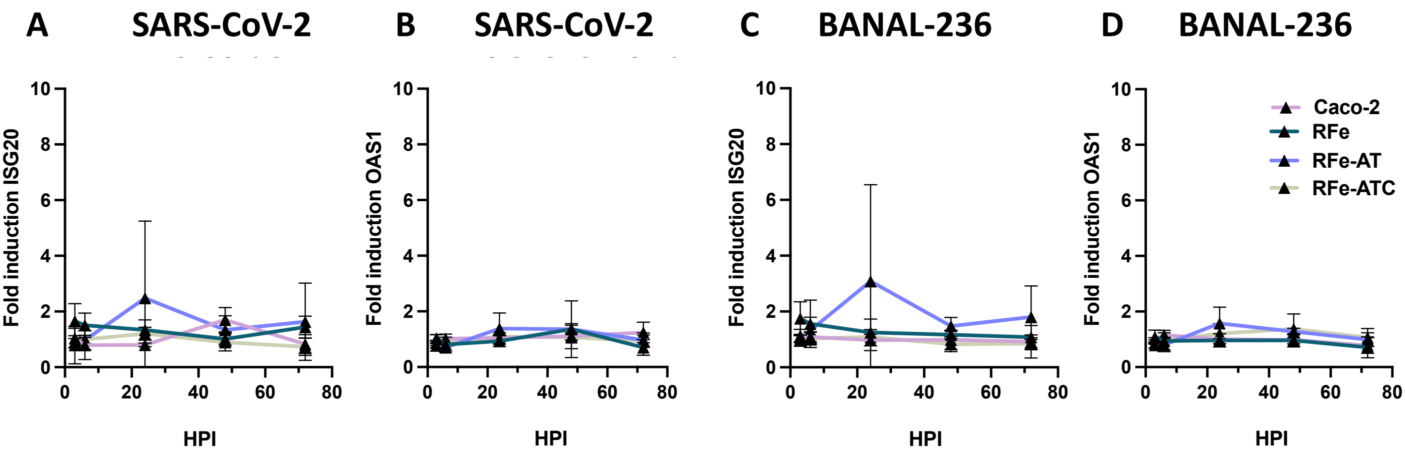

Figure S4. Interferon-stimulated genes are not induced upon SARS-CoV-2 and BANAL-236 replication in Caco-2 and RFe-ATC cells. RFe (green), RFe-AT (blue) and RFe-ATC (gray) cells were infected with SARS-CoV-2 at a multiplicity of infection (MOI) of 0.2 **(A, B)** or with BANAL-236 at an MOI of 0.5 **(C, D).** Caco-2 cells (pink), were infected with SARS-CoV-2 at an MOI of 0.0002 **(A, B)** or were infected with BANAL-236 at a MOI of 0.02 **(C, D).** Cell lysates were collected for RT-qPCR analysis at 3h, 6h, 24h, 48h, 72h. The relative amounts of ISG20 **(A, C)** and OAS1 **(B, D)** mRNAs were determined by RT-qPCR analysis. Results were first normalized to GAPDH mRNA and then to mRNA levels of mock-infected cells, which were set at 1. Data are means ± SD of at least two independent experiments.
