# Supplementary material for: Entry, replication and innate immunity evasion of BANAL-236, a SARS-CoV-2-related bat virus, in Rhinolophus and human cells
Source: PLoS Pathog. 2026 Apr 20;22(4):e1013573. doi: 10.1371/journal.ppat.1013573 (PMC13108884; doi:10.1371/journal.ppat.1013573)
Supplement: S5 Fig — (PDF) [file ppat.1013573.s005.pdf]

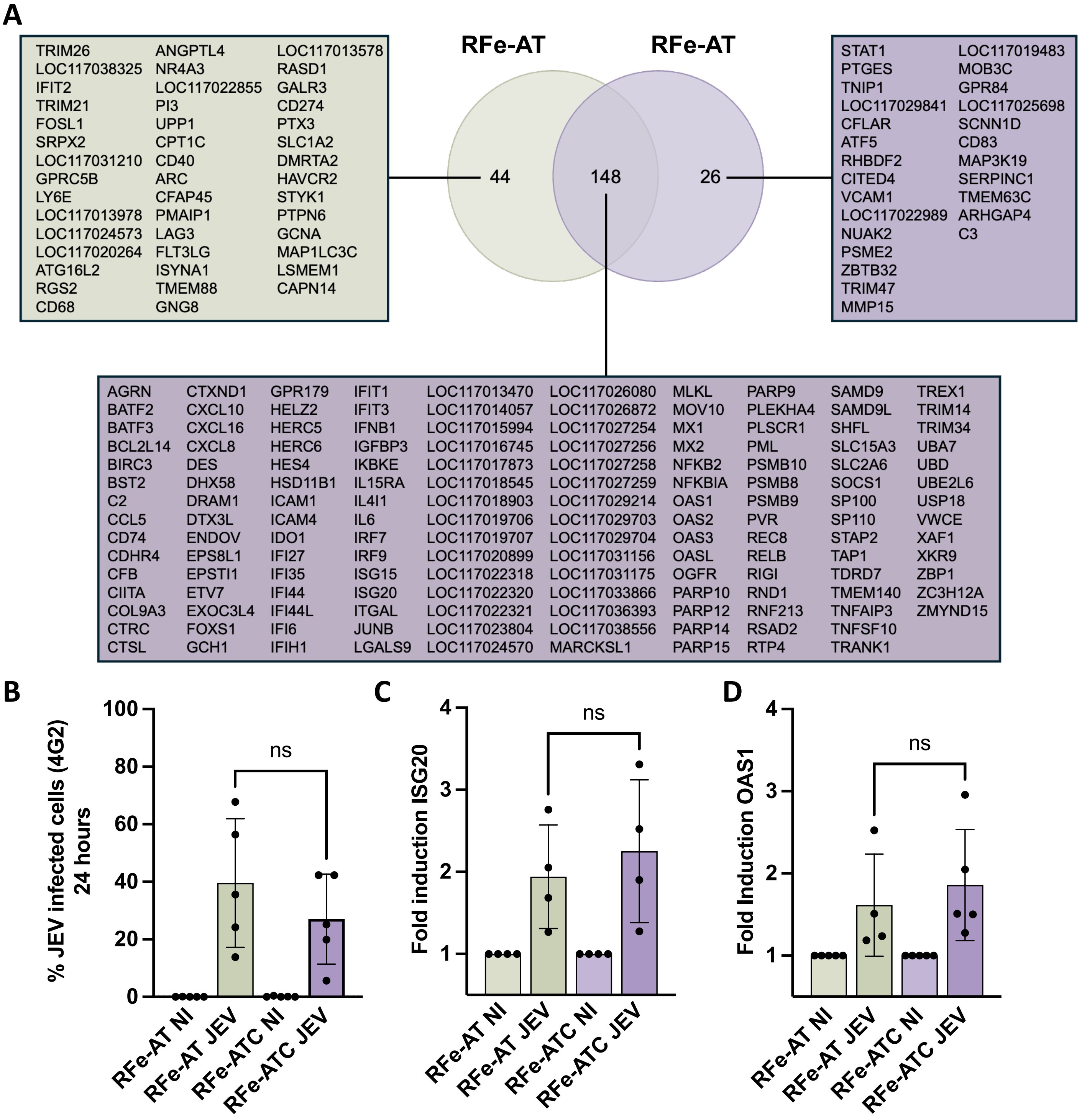

Figure S5. RFe-AT and RFe-ATC cells are immunocompetent. (A) Venn diagram representing the distribution and composition of upregulated genes in RFe-AT and RFe-ATC cells upon poly I:C treatment. **(B-D)** RFe-AT and RFe-ATC cell were infected with Japanese encephalitis virus (JEV) at and MOI of 10. **(B)** The percentage of infected cells at 24 hours post-infection was assessed by staining the viral E protein by flow cytometry analysis using a pan-flavivirus 4G2 antibody. Data are means ± SD of at least three independent experiments. One-way ANOVA tests with Tukey's correction were performed. ns : non-significant. The relative amounts of ISG20 **(E)** and OAS1 **(F)** mRNAs after JEV infection were determined by RT-qPCR analysis. Results were first normalized to GAPDH mRNA and then to mRNA levels of control non infected cells. Data are means ± SD of at least three independent experiments. One-way ANOVA tests with Tukey's correction were performed. ns : non-significant.
