# Supplementary material for: Entry, replication and innate immunity evasion of BANAL-236, a SARS-CoV-2-related bat virus, in Rhinolophus and human cells
Source: PLoS Pathog. 2026 Apr 20;22(4):e1013573. doi: 10.1371/journal.ppat.1013573 (PMC13108884; doi:10.1371/journal.ppat.1013573)
Supplement: S2 Table — (PPTX) [file ppat.1013573.s007.pptx]

## Slide 1
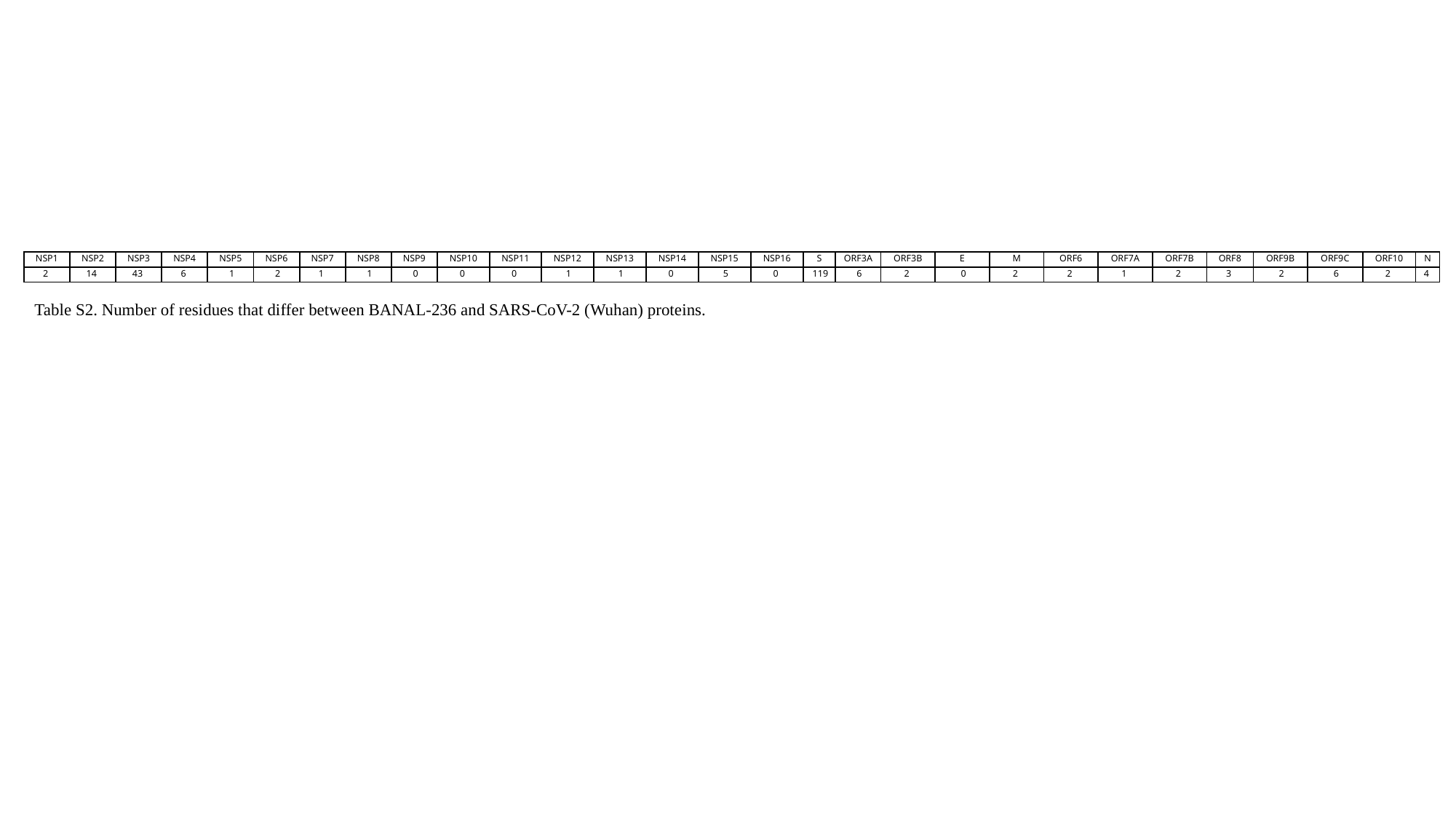

| NSP1 | NSP2 | NSP3 | NSP4 | NSP5 | NSP6 | NSP7 | NSP8 | NSP9 | NSP10 | NSP11 | NSP12 | NSP13 | NSP14 | NSP15 | NSP16 | S | ORF3A | ORF3B | E | M | ORF6 | ORF7A | ORF7B | ORF8 | ORF9B | ORF9C | ORF10 | N |
| --- | --- | --- | --- | --- | --- | --- | --- | --- | --- | --- | --- | --- | --- | --- | --- | --- | --- | --- | --- | --- | --- | --- | --- | --- | --- | --- | --- | --- |
| 2 | 14 | 43 | 6 | 1 | 2 | 1 | 1 | 0 | 0 | 0 | 1 | 1 | 0 | 5 | 0 | 119 | 6 | 2 | 0 | 2 | 2 | 1 | 2 | 3 | 2 | 6 | 2 | 4 |
Table S2. Number of residues that differ between BANAL-236 and SARS-CoV-2 (Wuhan) proteins.
