# Supplementary material for: Entry, replication and innate immunity evasion of BANAL-236, a SARS-CoV-2-related bat virus, in Rhinolophus and human cells
Source: PLoS Pathog. 2026 Apr 20;22(4):e1013573. doi: 10.1371/journal.ppat.1013573 (PMC13108884; doi:10.1371/journal.ppat.1013573)
Supplement: S3 Table — (DOCX) [file ppat.1013573.s008.docx]

| **Plasmid** | **Template** | **Primers** |
| --- | --- | --- |
| pDONR221-BANAL-236-NSP1 | BANAL-236 cDNA | For : 5’- GGGGACAAGTTTGTACAAAAAAGCAGGCTCAATGGAGAGCCTTGTCCCTGG  Rev : 5’- GGGGACCACTTTGTACAAGAAAGCTGGGTGTTATCCTCCGTTAAGCTCACGCA |
| pDONR221-BANAL-236-NSP2 | BANAL-236 cDNA | For : 5’- GGGGACAAGTTTGTACAAAAAAGCAGGCTCAATGGCATACACTCGCTATGTCGAT  Rev : 5’- GGGGACCACTTTGTACAAGAAAGCTGGGTGTTAACCGCCTTTAAGTGTGAAGGT |
| pDONR221-BANAL-236-NSP4 | BANAL-236 cDNA | For : 5’- GGGGACAAGTTTGTACAAAAAAGCAGGCTCAATGAAAATTGTTAATAACTGGTTGAAG  Rev : 5’- GGGGACCACTTTGTACAAGAAAGCTGGGTGTTACTGCAAAACAGCTGAGGTGA |
| pDONR221-BANAL-236-NSP12 | BANAL-236 cDNA | For : 5’- GGGGACAAGTTTGTACAAAAAAGCAGGCTCAATGAGTGCAGCCCGTCTTACACC  Rev : 5’- GGGGACCACTTTGTACAAGAAAGCTGGGTGTTACTGTAAGACTGTATGGGGTG |
| pDONR221-BANAL-236-NSP15 | BANAL-236 cDNA | For : 5’- GGGGACAAGTTTGTACAAAAAAGCAGGCTCAATGAGTTTGGAAAATGTGGCTTTT  Rev : 5’- GGGGACCACTTTGTACAAGAAAGCTGGGTGTTATTGTAATTTTGGGTAAAATGT |
| pDONR221-BANAL-236-N | BANAL-236 cDNA | For : 5’- GGGGACAAGTTTGTACAAAAAAGCAGGCATGTCTGATAATGGACCCCAA  Rev : 5’- GGGGACCACTTTGTACAAGAAAGCTGGGTGTTAGGCCTGAGTTGAATCAGCAC |
| pDONR221-BANAL-236-ORF3a | BANAL-236 cDNA | For : 5’- GGGGACAAGTTTGTACAAAAAAGCAGGCTCAATGGATTTGTTTATGAGAAT  Rev : 5’- GGGGACCACTTTGTACAAGAAAGCTGGGTGTTACAAAGGCACGCTAGTAGTCG |
| pDONR221-BANAL-236-ORF3d | BANAL-236 cDNA | For : 5’- GGGGACAAGTTTGTACAAAAAAGCAGGCTCAATGGCTTATTGTTGGCGTTG  Rev : 5’- GGGGACCACTTTGTACAAGAAAGCTGGGTGTTAAGGCCAGCAGCAACGAGCAA |
| pDONR221-BANAL-236-ORF6 | BANAL-236 cDNA | For : 5’- GGGGACAAGTTTGTACAAAAAAGCAGGCATGTTTCATCTCGTTGACTT  Rev : 5’- GGGGACCACTTTGTACAAGAAAGCTGGGTGTTAATCAATCTCCATTGGTTGCTCT |
| pDONR221-BANAL-236-ORF9b | BANAL-236 cDNA | For : 5’- GGGGACAAGTTTGTACAAAAAAGCAGGCTCAATGGACCCCAAAATCAGCGA  Rev : 5’- GGGGACCACTTTGTACAAGAAAGCTGGGTGTTATTTTACCGTCACCACCACGA |
| pDONR221-BANAL-236-ORF9c | BANAL-236 cDNA | For : 5’- GGGGACAAGTTTGTACAAAAAAGCAGGCTCAATGCTGCAATCGTGCTACAAC  Rev : 5’- GGGGACCACTTTGTACAAGAAAGCTGGGTGTTAATCTGTCAAGCAGCAGCAAA |
| P3XFLAG-CMV-10-BANAL NSP3 | BANAL_236 cDNA | For : 5’- ATCAAGCGGCCGCGGCACCAACGAAAGTTACTTTTGGC  Rev : 5’- ATGCTTGGATCCTTAACCACCCTTGAGTGCTATCTTTG |
| **Plasmid** | **Template** | **Sequences** |
| pDONR221-BANAL-236-NSP11 | synthetic | 5’-GGGGACAAGTTTGTACAAAAAAGCAGGCATGTCAGCTGATGCACAATCGTTTTTAAACGGGTTTGCGGTGTAA  CACCCAGCTTTCTTGTACAAAGTGGTCCCC |
| pDONR221-BANAL-236-ORF3d | synthetic | 5’-GGGGACAAGTTTGTACAAAAAAGCAGGCATGGCCTATTGTTGGAGGTGTACCTCTTGCTGCTTCTCCAAGAGG  TTCCAGAACCACAACCCCTAACACCCAGCTTTCTTGTACAAAGTGGTCCCC |
| pDONR221-BANAL-236-ORF6 | synthetic | 5’GGGGACAAGTTTGTACAAAAAAGCAGGCATGTTCCACCTGGTGGATTTCCAGGTGACTATCGCCGAGATCCTGCTGATCATCATGCGGACCTTTAAGGTGAGCATCTGGAACTTCGACTACATCATTAATCTGATCATCAAGAACCTGTCCAAGAGCCTGACCGAGAACAAGTACTCTCACCTGGATGAGGAGCAGCCAATGGAAATCGACTAACACCCAGCTTTCTTG  TACAAAGTGGTCCCC |
| pDONR221-BANAL-236-ORF9c | synthetic | 5’GGGGACAAGTTTGTACAAAAAAGCAGGCATGCTGCAATCGTGCTACAACTTCCTCAAGGAACAACATTGCCAAAAGGCTTCTACGCAGAGGGAAGCAGGGGCGGCAGTCAAGCTTCTTCTCGCTCCTCATCACGTAGTCGCAACAGTTCAAGAAATTCAACTCCAGGCAGCAGTAGGGGAACTTCTCCTGCTAGGATGGCTGGCAATGGCGGTGATGCTGCTCTTGCTTTGCTGCTGCTTGACAGATTAACACCCAGCTTTCTTGTACAAAGTGGTCCCC |
| pDONR221-BANAL-236-ORF10 | synthetic | 5’-GGGGACAAGTTTGTACAAAAAAGCAGGCATGGGCTACATCAACGTGTTCGCCAGCCCTTTCACCATCTACAG  CCTGCTGCTGTGCAGAATGAATAGCAGAAGCTACATCGCCCAGGTGGACGTGGTGAACTTTAATCTGACTAACACCCAGCTTTCTTGTACAAAGTGGTCCCC |
| pLVX-EF1alpha-  SARS-BANAL-236-orf8-STREP | synthetic | 5’GAATTCGCCGCCACCATGAAGTTCCTGGTGTTTCTGGGCATTCTGACCACCGTGGCCGCCTTTCACCAGGAATGCTCCCTGCAGAGCTGTGCCCAGCACCAGCCCTACGTGGTGGATGACCCTTGCCCAATCCATTTCTACAGCAAATGGTACATCAGGGTGGGCGCCAGAAAGAGCGCCCCACTGATTGAACTGTGCGTGGACGAGGCTGGCAGCAAGTCCCCCATCCAGTATATCGATATTGGCAACTACACCGTGTCCTGCTCACCCTTTACCATCAACTGCCAGGAGCCAAAGCTGGGCTCCCTGGTGGTGCGGTGTTCCTTCTACGAGGACTTCCTGGAGTATCATGATGTGCGGGTCGTGCTGGATTTCATCCTCGAAGGCGGCGGGGGATGGAGCCATCCACAATTCGAGAAAGGCGGTGGTTCAGGAGGAGGTAGCGGGGGTGGATCATGGTCACATCCGCAGTTTGAAAAGTAAGGATCC |
| pLVX-EF1alpha-  SARS-BANAL-236-S-STREP | synthetic | 5’GAATTCGCCGCCACCATGCTGTTTTTTTTCTTCCTGTGTTTCGCCTCCGTGAACAGCCAGTGCGTGAATCTCACCGGTAGAGCAACCATCCAGCCATCTTTTACTAATTCTAGCCACAGGGGAGTGTACTACCCCGATACCATCTTCCGCTCCAACAGCCTGGTCCTTTCTCAGGGCTATTTTCTGCCCTTCTACAGTAACATCTCCTGGTACTACGCCCTGACCAAAACCAACGGCGCCGAGAAAAGAGTCGATAATCCCATCCTGGATTTCAAAGACGGCATCTATTTCGCCGCAACTGAAAAGAGCAACATTGTGAGGGGCTGGATCTTCGGAACAACCCTGGACAATACATCCCAGTCTCTGCTGATCGTGAACAACGCCACCAACGTGATCATTAAGGTGTGCAACTTTCAGTTTTGCTATGATCCATACCTGAGTGGCTACTTCCACAATAATAAGACTTGGAGCACAAGAGAGTTTGCCGTCTACAGCAGTTACGCCAATTGCACATTCGAATATGTGAGCAAGCCATTCATGCTGGACATCAGTGGGAAGAGTGGATTATTTGACACACTGAGAGAGTTCGTGTTCCGGAACGTGGACGGATACTTTAAGATCTACAGCAAGTATAGCCCTGTGAATGTGAACAGCAACCTGCCTAGTGGGTTCAGCGCCCTGGAGCCACTGGTCGAGTTGCCCGCCGGCATTAATATCACCCGCTTCAGAACCCTCCTGACAATCCATAGGGGCGACCCAATGCCTAACAATGGGTGGACCGTGTTTAGCGCCGCTTACTATGTGGGGTACCTGGCCCCAAGGACATTCATGCTGAAGTATAACGAAAATGGCACCATCACTGACGCCGTTGACTGTTCCCTGGACCCCCTGTCAGAGGCCAAGTGCACTCTGAAGTCATTCACCGTGGAGAAGGGCATCTACCAGACATCTAACTTCAGAGTGCAGCCTACCGATAGCATCGTGCGCTTCCCAAATATTACCAATCTGTGTCCATTCGGCGAGGTGTTCAACGCCACCACATTCGCCAGCGTGTACGCCTGGAATCGCAAGAGGATCAGCAACTGTGTGGCCGACTATTCCGTGCTGTACAATAGCACCTCTTTCAGCACCTTTAAATGCTACGGCGTGAGCCCAACTAAGCTGAATGACCTGTGTTTCACAAACGTGTATGCCGATTCCTTCGTGGTGCGGGGGGATGAGGTGAGGCAGATCGCCCCTGGCCAGACCGGCAAGATCGCCGACTATAACTACAAGCTGCCCGATGACTTCACTGGATGCGTGATCGCCTGGAATAGCAACAATCTCGACTCTAAGGTGGGGGGCAATTACAATTATCTGTACAGGCTGTTCAGGAAATCTAACCTGAAACCATTCGAAAGAGACATTAGCACCGAGATTTACCAGGCTGGCTCTACTCCATGTAATGGAGTGGAGGGGTTCAATTGCTACTTTCCCCTGAAGAGCTACGGATTTCACCCCACCAATGGCGTGGGATATCAGCCTTACCGAGTGGTGGTGCTGAGCTTCGAGCTGCTGAACGCCCCCGCCACCGTGTGTGGCCCAAAGAAGAGTACCAACCTGATCAAGAATAAGTGCGTGAATTTTAATTTCAACGGCCTGACAGGAACCGGCGTGCTGACTGAGAGCAACAAGAAGTTCCTGCCTTTCCAGCAGTTTGGGAGAGATATCGCTGATACCACCGATGCAGTGCGCGACCCTCAGACCCTGGAGATCCTGGATATTACCCCCTGCAGTTTCGGCGGCGTGAGCGTGATCACACCTGGCACCAATGCAAGCAACCAGGTGGCCGTGCTATACCAGGATGTGAATTGCACTGAAGTTCCAGTGGCCATCCACGCCGACCAGCTGACCCCTACCTGGAGAGTGTACAGCACCGGGTCAAATGTCTTCCAGACAAGAGCCGGCTGTCTGATCGGCGCCGAGCACGTCAATAATAGCTACGAGTGTGATATCCCCATCGGGGCAGGAATCTGTGCCAGCTACCAGACGCAGACCAATTCTAGAAGCGTGGCCTCCCAGAGTATCATCGCCTATACCATGAGCCTTGGGGCCGAGAACAGCGTGGCTTATAGCAATAACAGCATTGCCATTCCCACAAACTTCACCATCTCTGTGACCACCGAAATCCTGCCAGTGTCAATGACCAAGACTTCAGTGGATTGTACCATGTACATCTGCGGCGATAGCACAGAGTGTTCCAACCTGCTGTTGCAGTACGGCTCCTTCTGCACCCAGCTGAACAGGGCCCTGACCGGGATTGCCGTGGAACAGGACAAAAATACACAGGAAGTGTTCGCTCAGGTGAAGCAGATCTACAAAACCCCACAGATCAAGGATTTCGGCGGCTTTAACTTCTCCCAGATCCTGCCAGATCCTAGCAAGCCCAGCAAGAGGAGCTTCATCGAGGATCTGCTGTTCAATAAGGTGACCCTGGCAGACGCCGGTTTCATCAAGCAATACGGCGACTGCCTGGGAGACATCGCCGCTAGAGACCTGATCTGCGCCCAGAAGTTCAATGGCCTGACAGTGCTGCCCCCTCTGCTGACCGATGAGATGATCGCTCAGTACACCTCCGCTCTGCTGGCAGGCACCATCACAAGTGGTTGGACCTTTGGTGCCGGTGCCGCCCTGCAGATTCCCTTTGCTATGCAGATGGCCTACCGCTTTAACGGTATCGGCGTGACCCAGAACGTGCTGTACGAGAACCAGAAACTGATTGCCAACCAGTTTAACTCCGCCATCGGCAAAATACAGGACAGCCTCAGTAGCACCGCCTCGGCCCTTGGCAAGCTGCAGGACGTGGTGAATCAGAACGCTCAGGCCCTGAATACACTGGTGAAGCAGCTGAGCTCAAATTTTGGGGCCATATCTAGCGTGCTGAATGACATCCTGTCCAGACTGGACAAGGTGGAGGCAGAGGTGCAGATTGATCGCCTGATTACCGGGAGACTGCAAAGCCTCCAGACCTACGTGACCCAGCAGCTCATCAGAGCCGCAGAAATAAGAGCCTCCGCCAATCTGGCCGCCACAAAAATGTCCGAATGTGTGCTGGGGCAGAGCAAGCGCGTGGACTTTTGTGGCAAGGGCTACCACCTGATGAGCTTCCCGCAGTCCGCCCCTCATGGCGTGGTCTTCCTCCACGTGACCTATGTCCCTGCCCAAGAAAAAAATTTCACCACCGCCCCTGCCATCTGCCACGACGGCAAGGCACATTTTCCCAGGGAGGGAGTGTTTGTGAGCAACGGAACCCACTGGTTCGTGACCCAGAGGAATTTTTACGAGCCACAGATTATCACCACAGATAACACCTTCGTTAGTGGAAACTGCGACGTCGTGATTGGCATCGTGAATAACACCGTGTACGACCCTTTGCAGCCCGAGCTGGATAGCTTTAAGGAAGAGCTGGACAAATACTTCAAGAACCACACCTCTCCTGATGTGGACCTGGGGGACATCAGCGGAATCAACGCCAGCGTGGTGAATATCCAGAAGGAGATCGACAGACTGAATGAGGTGGCCAAGAACCTGAATGAGAGCCTGATCGACCTGCAGCAGCTGGGGAAGTATGAGCAGTATATCAAGTGGCCATGGTACATCTGGCTGGGCTTCATCGCTGGCCTGATCGCCATCGTCATGGTGACCATCATGCTGTGCTGCATGACATCTTGCTGTAGCTGCCTGAAGGGCTGTTGCTCATGTGGCTCCTGCTGCAAGTTTGATGAAGACGACTCTGAGCCTGTGCTGAAAGGCGTCAAGCTGCACTACACACTCGAAGGCGGCGGGGGATGGAGCCATCCACAATTCGAGAAAGGCGGTGGTTCAGGAGGAGGTAGCGGGGGTGGATCATGGTCACATCCGCAGTTTGAAAAGTAAGGATCC |

**Table S3.** Primers and sequences used for cloning BANAL-236 ORFs.
