# Supplementary material for: Entry, replication and innate immunity evasion of BANAL-236, a SARS-CoV-2-related bat virus, in Rhinolophus and human cells
Source: PLoS Pathog. 2026 Apr 20;22(4):e1013573. doi: 10.1371/journal.ppat.1013573 (PMC13108884; doi:10.1371/journal.ppat.1013573)
Supplement: S4 Table — (DOCX) [file ppat.1013573.s009.docx]

| **Plasmid** | **Template** | **Primers** |
| --- | --- | --- |
| pDONR07-BANAL-236-NSP5 | pDONOR207-SARS-CoV-2-NSP5 | For : 5’- actgacctggagggtgccttctacggccccttc  Rev : 5’- gaaggggccgtagaaggcaccctccaggtcagt |
| pDONR207-BANAL-236-NSP6 | pDONR207-RaTG13-NSP6 | For : 5’- acaagcctgagcggcctgaagctgaaggactgc  Rev : 5’- gcagtccttcagcttcaggccgctcaggcttgt |
| pDONR207-BANAL-236-NSP7 | pDONR207-RaTG13-NSP7 | For : 5’- gcagggcgccgtggacatttctaagctgtgcgaggaaat  Rev : 5’- atttcctcgcacagcttagaaatgtccacggcgccctgc |
| pDONR207-BANAL-236-NSP8 | pDONR207-RaTG13-NSP8 | For : 5’- ctgaatatcatccctctgattaccgccgccaagctgatg  Rev : 5’- catcagcttggcggcggtaatcagagggatgatattcag |
| pDONR207-BANAL-236-NSP13 | pDONR207-SARS-CoV-2-NSP13 | For : 5’- actacgtgcgcatcatcggactgtacccaac  Rev : 5’- gttgggtacagtccgatgatgcgcacgtagt |
| pDONR207-BANAL-236-M | pDONR207-RaTG13-M | For : 5’- ggcaccatcaccgtggacgaactgaagaaactg  Rev : 5’- cagtttcttcagttcgtccacggtgatggtgcc |
| pDONR207-BANAL-236-ORF7a | pDONR207-SARS-CoV-2-orf7a | For : 5’- gccccatcttcctgatcattgccgctatcgtgttcat  Rev : 5’- atgaacacgatagcggcaatgatcaggaagatggggc |
| pDONR207-BANAL-236-ORF7b | pDONR207-RaTG13-orf7b | For : 5’- ctggccttcctgctgctgctggtgctgattatgc  Rev : 5’- gcataatcagcaccagcagcagcaggaaggccag |

**Table S4.** Primers used for mutagenesis.
