# Supplementary material for: Entry, replication and innate immunity evasion of BANAL-236, a SARS-CoV-2-related bat virus, in Rhinolophus and human cells
Source: PLoS Pathog. 2026 Apr 20;22(4):e1013573. doi: 10.1371/journal.ppat.1013573 (PMC13108884; doi:10.1371/journal.ppat.1013573)
Supplement: S5 Table — (DOCX) [file ppat.1013573.s010.docx]

| **Antibodies** | **Species** | **Dilution WB** | **Dilution IF/FC** | **Reference** | **Supplier** |
| --- | --- | --- | --- | --- | --- |
| **Primary antibodies** |  |  |  |  |  |
| Anti-ACE2 | Rabbit | 1:2000 | 1:500 | AB15348 | Abcam |
| Anti-TMPRSS2 | Fc human |  | 1:900 | VHH-A01 | O. Schwart Lab, Institut Pasteur, Paris Saunders and al (2023) |
| Anti-TMPRSS2 | Rabbit | 1:1 000 |  | HPA035787 | Atlas |
| Anti-C-myc | Rabbit | 1:1 000 | 1:200 | AB32072 | Abcam |
| Anti-Flavivirus enveloppe 4G2 | Mouse |  | 1:1 000 | D1-4G2-4-15 (4G2) | (kind gift from Philippe Desprès |
| Anti-NCp15 |  |  | 1:10 000/1:250 |  | CEA / O. Schwart Lab, Institut Pasteur, Paris Planas and al (2023) |
| Anti-Strep-tag | Mouse | 1:1000 |  | 34850 | qiagen |
| Anti-Flag | Mouse | 1:4000 |  | F3165-1MG | Sigma Aldrich |
| Anti-V5 | Mouse | 1:5 000 | 1:200 | 46-0705 | Thermo Fisher Scientific |
| Anti-IFITM3 | Rabbit |  | 1:200 | PA511274 | Invitrogen |
| anti-actin | Mouse | 1: 10 000 |  | A5316 | Sigma Aldrich |
| Anti-GAPDH | Rabbit |  | 1:5 000 | 10494-1-AP | Proteintech |
| anti-hACE2-647 | Goat | 1:300 | 1:250 | FAB933R | R&D Systems |
| Anti-Strep-tag | Mouse | 1:1000 |  | 34850 | qiagen |
| **Secondary antibodies** |  |  |  |  |  |
| Alexa Fluor 488 anti-mouse IgG (H+L) | Goat | - | 1:1 000 | A11001 | Life Technologies |
| Alexa Fluor 647 anti-rabbit IgG (H+L) | Donkey | - | 1:1 000 | A-31573 | Life Technologies |
| Alexa Fluor 680 anti-mouse IgG (H+L) | Goat | 1:10 000 | 1:1 000 | A-21057 | Invitrogen |
| Anti-rabbit IgG (H+L) Dylight 800 | Goat | 1:10 000 | 1:1 000 | SA5‐35571 | Invitrogen |
| Alexa Fluor 647 anti-human IgG (H+L) | Goat | 1:10 000 | 1:1 000 | A-21445 | Invitrogen |

**Table S5.** Antibodies used in the study.
