# Supplementary material for: Entry, replication and innate immunity evasion of BANAL-236, a SARS-CoV-2-related bat virus, in Rhinolophus and human cells
Source: PLoS Pathog. 2026 Apr 20;22(4):e1013573. doi: 10.1371/journal.ppat.1013573 (PMC13108884; doi:10.1371/journal.ppat.1013573)
Supplement: S6 Table — (DOCX) [file ppat.1013573.s011.docx]

| **Target gene** | **Forward Primer 5'-->3'** | **Reverse Primer 5'-->3'** |
| --- | --- | --- |
| hGAPDH | GGTCGGAGTCAACGGATTTG | ACTCCACGACGTACTCAGCG |
| rGAPDH | GACAACTTCGGCATCGTGGA | TGCGAGTGAGCTTTCCATTGA |
| hACE2 | GGACCCAGGAAATGTTCAGA | GGCTGCAGAAAGTGACATGA |
| rACE2 | TGACTGGGTTTTGAACAGTGCC | TGGATTGAGCAGCGGTTACA |
| hTMPRSS2 | CAAGTGCTCCAACTCTGGGAT | AACACACCGATTCTCGTCCTC |
| rTMPRSS2 | CAAGTGCTCGGTGTCCGGGAT | AACGCACCGGTTCTCGTCCTC |
| hOAS1 | GAGCTCCTGACGGTCTATGC | TTCGTGAGCTGCCTTCTCAG |
| rOAS1 | CAAAGTCGTGAAGGGTGGCTC | AACTTCTGAGGTGGCTGAGG |
| hISG20 | TGGACTGCGAGATGGTGG | GGGTTCTGTAATCGGTGAA |
| rISG20 | TACAGAACCCGAGTCAGCGG | AGCGCCTTAAAGTCGTGTTTC |
| Pan-sarbecovirus E | ACAGGTACGTTAATAGTTAATAGCGT | ATATTGCAGCAGTACGACCACA |

**Table S6.** Primers used for qPCR amplification of Human and *Rhinolophus ferrumequinum* genes.
