# Supplementary material for: Entry, replication and innate immunity evasion of BANAL-236, a SARS-CoV-2-related bat virus, in Rhinolophus and human cells
Source: PLoS Pathog. 2026 Apr 20;22(4):e1013573. doi: 10.1371/journal.ppat.1013573 (PMC13108884; doi:10.1371/journal.ppat.1013573)
Supplement: S7 Table — (DOCX) [file ppat.1013573.s012.docx]

**Table S7.** Frequency of allelic mutations observed in BANAL-236 C2 and C3 passages performed in VeroE6 cells. Mutations are compared to the original BANAL-236 sequence (GenBank accession number MZ937003.2) obtained from bat rectal swab. Synonymous (S) and non-synonymous (NS) mutations, and only frequencies above 1% are reported.
