# Supplementary material for: Entry, replication and innate immunity evasion of BANAL-236, a SARS-CoV-2-related bat virus, in Rhinolophus and human cells
Source: PLoS Pathog. 2026 Apr 20;22(4):e1013573. doi: 10.1371/journal.ppat.1013573 (PMC13108884; doi:10.1371/journal.ppat.1013573)
Supplement: Attachment — Submitted filename: Rebuttal letter .docx [file ppat.1013573.s014.docx]

EDITOR COMMENTS:

I obtained two reviews from experts, and have also read the manuscript carefully myself. The reviewers had many critiques, most of which I concur with. This paper does have a lot of interesting findings including looking at aspects of replication in bat versus human cells, but many of the claims are overstated. I am willing to consider a revised version of the manuscript, but only if it makes large-scale changes to address the reviewer comments as well as my own comments which I have put directly below. This will require substantial revision, and please only submit a revised version if you can address the main points and much more clearly not make over-claims related to points that cannot be fully addressed.

MAJOR COMMENTS:

- The manuscript contains numerous statements suggesting that BANAL-236 is more efficient at using human ACE2 than Rhinolophus ACE2. As also pointed out by reviewer 1, these statements are not justified and given the wrong impression. Rhinolophus bat ACE2 is known to be under positive selection for variation in the region bound by sarbecovirus spikes, such that different Rhinolophus alleles can have dramatically different abilities to support cell entry (eg, see Guo et al, Journal Virology, 94:e00902-20; Starr et al, Nature, 603:913; Li et al, PLoS Pathogens, 10.1371/journal.ppat.1011116; and many other papers). Furthermore, there is no reason to think that sequencing of Rhinolophus bat ACE2s is anywhere close to exhaustively sampling all the natural alleles, and further this paper did not test most of the known alleles. Therefore, to imply that BANAL-236 uses human ACE2 more efficiently than Rhinolophus ACE2 is not supported without more exhaustively testing Rhinolophus ACE2s. So the paper should not claim as a conclusion anything about ACE2 usage, rather this is more like an experimental limitation that only limited testing of different Rhinolophus ACE2 alleles was done.

We have revised the text to emphasize the experimental limitation of our entry assays in the discussion, explicitly stating that further testing of diverse *Rhinolophus* ACE2 variants is needed to draw definitive conclusions (lines 474-77 and lines 484-85). Additionally, we added some references to provide context on the known variability in *Rhinolophus* ACE2 and its implications for sarbecovirus entry.

Lines 474-77:

*‘That said, the results of the entry assays must be interpreted cautiously. Our study used only one R. malayanus ACE2 sequence, yet Rhinolophus ACE2 alleles are known to exhibit substantial variabilities in their ability to support viral entry [55–57]. Furthermore, current sequencing efforts have not exhaustively sampled the full diversity of Rhinolophus ACE2 alleles.’*

Lines 484-85:

*‘….future studies should systematically evaluate the binding affinity of their* ***RBDs*** *to* ***ACE2*** *from* ***a comprehensive collection of alleles from different Rhinolophus species****. Such approach would enable definitive conclusions about host range and ACE2 usage.’*

- What controls were performed to ensure that the epitope tags introduced onto the bat ACE2 and TMPRSS2 did not interfere with their functions as receptors and spike activating proteases?

Of note, the entry assays shown in panels 2A-E were performed on 293T cells expressing V5-tagged versions of human or *Rhinolophus* ACE2. Similarly, both human and *Rhinolophus* TMPRSS2 were N-terminally myc-tagged. We have made it clearer in the main text and in the MM section that the human and bat proteins were tagged in the same manner (lines 177-79 and 642-45). Additionally, we have modified the axis labels in panels 2A and 2B, and the legend of figure 2 to reflect this. Western blots showing the expression of hACE2-V5, rACE2-V5, myc-hTMPRSS2 and myc-rTMPRSS2 in transfected 293T cells, detected using antibodies against hACE2, hTMPRSS2, V5 or myc, have been added as supplemental panels (novel panels S1A and B).

To assess whether the V5 tag interferes with ACE2 receptor function, we performed SARS-CoV-2 infection experiments in A549 cells. SARS-CoV-2 does not replicate in wild-type A549 cells unless they express hACE2 (DOI: [10.1128/jvi.00608-22](https://doi.org/10.1128/jvi.00608-22) ). We established A549 cells stably expressing hACE2-V5 or rACE2-V5 (novel panel S1D). Flow cytometry analysis using an anti-S antibody at 24 hours post-infection (MOI 1) revealed that 15% of hACE2-V5-expressing cells were positive for the S protein (novel panel S1E), indicating that viral S protein binds hACE2-V5. Our previous experiments showed that around 3% of A549 cells expressing untagged hACE2 were expressing the SARS-CoV-2 S proteins after 24 hours of infection with the same MOI (DOI: [10.1128/jvi.00608-22](https://doi.org/10.1128/jvi.00608-22) ). Thus, the V5 tag does not interfere with hACE2 receptor function.

Of note, and as expected, expression of rACE2-V5 did not support SARS-CoV-2 replication in A549 cells, which aligns with our entry assays in 293T cells (Fig. 2E) and BHK-21 cells (novel Fig. S2E), where rACE2-V5 did not permit entry of lentiviruses bearing the SARS-CoV-2 S protein.

Since hACE2 is a known substrate of hTMPRSS2 (<https://doi.org/10.1128/jvi.02062-10>; <https://doi.org/10.1128/jvi.02202-13>), we performed Western blot analysis to determine whether myc-tagged hTMPRSS2 and rTMPRSS2 retain their ability to cleave V5-tagged ACE2. 293T cells were transfected with hACE2 and increasing amount of myc-TMPRSS2. In the absence of the protease, hACE2-V5 appeared as 2 bands of about ∼125-kDa (novel Fig. S1F), likely representing differentially glycosylated forms. In the presence of myc-hTMPRSS2, the heavier form of hACE2-V5 was not detectable anymore, while the ∼120-kDa form remained visible. Additionally, a ∼20-kDa ACE2 fragment appeared (Fig. S1F), consistent with cleavage by myc-hTMPRSS2. These results align with previous studies performed in 293T cells expressing untagged hACE2 and hTMPRSS2 (<https://doi.org/10.1128/jvi.02062-10>; <https://doi.org/10.1128/jvi.02202-13>). Identical hACE2 patterns were observed with myc-rTMPRSS2 (Fig. S1F). Together, these results indicate that myc-tagged human and *Rhinolophus* TMPRSS2 retain protease activity and efficiently process hACE2.

In summary, these results show that the V5-tagged hACE2 retains its receptor function for SARS-CoV-2 entry, and the myc-tagged h/r-TMPRSS2 maintain their protease activity, as evidenced by ACE2 cleavage. Therefore, the epitope tags do not seem to interfere with the functions of ACE2 or TMPRSS2 in our assays.

- Overall, as noted by both reviewers, while this study has interesting findings the limitations associated with using a single Rhinolophus cell line to make overly broad claims should be better described throughout the manuscript.

We have tempered our conclusions and emphasized that most of our findings are associated with the use of a single *Rhinolophus* cell line. For instance, lines 507-509:

*‘Thus, further investigations are needed to study BANAL-CoVs replication in bat cells derived from multiple tissues, including gastrointestinal tissues, particularly from Southeast Asian Rhinolophus species, such as R. marshalli and R.malayanus’.*

To investigate the susceptibility of other *Rhinolophus* cells, we performed additional experiments using immortalized *R. ferrumequinum* lung cells (RhiFLu), which were generated by the lab of Marcel Müller at Charité University in Berlin. These cells have not been published yet. We infected RhiFLu cells with increasing MOI of either SARS-CoV-2 or BANAL-236 for 24 or 48 hours (see rebuttal figure 1). Caco-2 cells, which are susceptible to both viruses (Fig. 1), served as positive controls. No N-positive cells were detected by flow cytometric analysis. We also generated RhiFLu cells stably expressing V5-tagged versions of hACE2 or rACE2, or GFP as a control. None of these cell lines were permissive to infection by either virus (Rebuttal figure 1). Therefore, at least 2 *Rhinolophus* cell lines are refractory to BANAL-236 replication, even when hACE2 is expressed: RFe and RhiFlu cells.

In addition, we have previously shown that primary fibroblast-like cells derived from *Rhinolophus ferrumequinum* patagium were not permissive to SARS-CoV-2 replication (DOI: [10.1128/jvi.00608-22](https://doi.org/10.1128/jvi.00608-22) ). Similarly, lung and [kidney cells](https://www.sciencedirect.com/topics/agricultural-and-biological-sciences/kidney-cell) from *R. sinicus* do not support SARS-CoV-2 replication ([10.1016/S2666-5247(20)30004-5](https://doi.org/10.1016/S2666-5247(20)30004-5), [0.3201/eid2612.202308](https://doi.org/10.3201/eid2612.202308)). Taken together, these results indicate that multiple *Rhinolophus* cells are refractory sarbecovirus replication. This point is now discussed in the main text (lines 441-445).

MINOR COMMENTS:

- Line 42: might clarify that you mean isolated directly from a primary sample, as other related viruses have been made by reverse genetics.

This has been clarified by replacing (now line 43)

*‘Here, we investigated the replication of BANAL-236… ‘*

by

*‘Here, we investigated the replication of* ***an isolate of*** *BANAL-236…’*

- Abstract lines 62-64: says it is "essential" to study BANAL-236 replication in bat cells to identify factors that could affect zoonotic spillover. This does not really make sense: it's not clear how studying in bat cells is essential to identify mechanisms that facilitate zoonotic spillover to humans; studying in bat cells is important for understanding replication in the natural host. I'm not disagreeing that this study is interesting or important, but this statement does not seem supported so remove this overstated rationale.

The sentence

*‘To elucidate the mechanisms underlying viral tolerance and to identify factors that could facilitate zoonotic spillover, it is essential to study the replication of bat-borne viruses in relevant bat cellular models.’*

was replaced by

*‘To elucidate the mechanisms underlying viral tolerance and to identify factors that could facilitate zoonotic spillover, it is essential to study the replication of bat-borne viruses in relevant bat* ***and human*** *cellular models’*

- Line 77-78 of Introduction: the SARS-CoV-2 genome is highly recombinant with different closest relatives in different genomic regions, so rather than saying the viruses are "the closest known relatives" it should say something more like "are among the closest known relatives" as for instance there are parts of the genome where RmYN02 (and other viruses) are more similar. Likewise, in the next sentence SARS-CoV-2 is unlikely to have arisen through recombination of these exact viruses, it would be better to say more like "An ancestor of SARS-CoV-2 may have originated from recombination and evolution of close relatives of these viruses and other Rhinolophus sarbecoviruses."

The sentence

*‘Three of these viruses are the closest known relatives of the Wuhan strain of SARS-CoV-2’*

was replaced by:

*‘Three of these viruses are* ***among*** *the closest known relatives of the Wuhan strain of SARS-CoV-2’*

The sentence

*‘An ancestral SARS-CoV-2 may have originated from recombination events involving these viruses and other closely related Rhinolophus sarbecoviruses’*

*was replaced by*

*‘An ancestor of SARS-CoV-2 may have originated from recombination and evolution of close relatives of these viruses and other Rhinolophus sarbecoviruses’.*

- Line 84-85: should probably note that all other sarbecoviruses (not just the BANAL ones) lack the FCS.

This information was added (lines 85-87).

Line 154-155: says replication of BANAL-236 similar to SARS-CoV-2 in Caco-2 cells, but in fact replication clearly worse: more virus had to be added, increase in titer from first to second timepoint was slower, and fewer cells every became N positive. In fact, the rest of the paragraph makes exactly this point, so it's not clear why this sentence says the replication kinetics are similar when in fact they are clearly slower.

This statement was modified and now reads as (lines 153-155):

*‘The growth kinetics of BANAL-236 were slower than those of SARS-CoV-2 in Caco-2 cells, showing a less rapid increase in RNA yield from the first to the second time point (Fig. 1C).’*

**Reviewers' Comments:**

Reviewer's Responses to Questions

**Part I - Summary**
Please use this section to discuss strengths/weaknesses of study, novelty/significance, general execution and scholarship.

Reviewer #1: Recent virus discovery studies have identified close relatives of SARS-CoV-2 in bats across southeast Asia. BANAL-236 and BANL-52 are two viruses identified in R. marshalli and R.malayanus, respectively. In this study, Gracias and colleagues performed a series of in vitro based experiments characterizing receptor compatibility, cell entry and viral replication of these viruses in bat and human cell lines. The results show curiously limited bat receptor use, strong human receptor compatibility, and the ability of BANAL-236 to partially inhibit interferon responses. While the results are interesting and help provide insights into interferon antagonism by SARS-CoV-2-related bat viruses, this study lightly hand waves a fair amount of bat biology at the expense of more meaningful conclusions. Regardless of these technical limitations (they can be easily addressed), the findings are timely and of interest to the PLoS Pathogens audience.

Reviewer #2: Gracias et. al characterize the replication and innate immune evasion of BANAL-236, a SARS-CoV-2-related bat sarbecovirus in both human and a novel Rhinolophus fibroblast bat cell line. Using both full-length and pseudotyped BANAL-236 virus, the authors demonstrate that BANAL-236 preferentially uses human ACE2 and TMPRSS2 over bat orthologs, and antagonizes host innate immune response pathways, effectively highlighting the zoonotic potential of this SARS-CoV-2-related coronavirus. Considering the ability of these viruses to recombine in nature and potentially adapt to human hosts, the study is timely and of interest to the coronavirus community. Experiments are also excellently conducted and the quality of the data is superb. However, there is a lack of mechanistic depth in describing the innate immune evasion pathways employed by BANAL-236, and the clonally derived bat cell line model is limited in its overall utility for studying these pathways. There are several significant gaps in the study, and their absence diminishes the overall impact of this manuscript and it’s fit within this journal. Major revisions are required to substantiate the overall claims.

**Part II – Major Issues: Key Experiments Required for Acceptance**
Please use this section to detail the key new experiments or modifications of existing experiments that should be absolutely required to validate study conclusions.

Generally, there should be no more than 3 such required experiments or major modifications for a "Major Revision" recommendation. If more than 3 experiments are necessary to validate the study conclusions, then you are encouraged to recommend "Reject".

Reviewer #1: MAJOR:
1. BANAL-236 was identified in R. marshalli and BANAL-52 was identified in R.malayanus. While convenient for its accessibility, R. fer­rume­quinum is not even from the same continent. I understand cells and tissues from the true BANAL virus hosts are not readily available, but the text should at least highlight this limitation when interpreting the results.

We have emphasized this point in the discussion (lines 507-509)

*‘Thus, further investigations are needed to study BANAL-CoVs replication in bat cells derived from multiple tissues, including gastrointestinal tissues, particularly from Southeast Asian Rhinolophus species, such as R. marshalli and R.malayanus.’*

2. All of the BANAL viruses were identified in fecal samples, and basically every other bat sarbecovirus described to date was identified in rectal/anal swabs or fecal samples. Therefore, I do not understand the authors' decision to produce lung-derived fibroblast cell lines to study these viruses. The authors claim the Rfe lung fibroblast cells are a relevant model but given that this is not the correct host species or presumed target tissue, these claims on "relevance" should be tempered. Maybe these bat cells are more relevant than 293Ts, but then again, 293Ts and other standard lab lines are used in this study to show many of the points because the RFe cells have issues.

The assertions regarding the relevance of the RFe models were tempered. Of note, these cells were generated at the onset of the COVID-19 pandemic, prior to the discovery of BANAL-236, when SARS-CoV-2 was primarily regarded as a respiratory virus. In collaboration with others, we attempted to produce gut-derived RFe cells, but these efforts were consistently compromised by contamination with endogenous bacteria.

3. Several recent studies have demonstrated the majority of beta-coronaviruses exhibit fairly tight species specificity (PMID: 39402048, 35114688, 40436893, 35895696, 40934083, 20567988), and even sarbecoviruses identified in R. sinicus are not capable of using every ACE2 isoform from R. sinicus bats (PMID: 32699095, 37655938). The 293T-based over-expresion experiment in figure 2E shows that BANAL-52 can use ACE2 from its own species while BANAL-236 does not use the same ACE2 orthologue, further supporting the idea that BANAL viruses only poorly use other bat ACE2s. This manuscript then claims BANAL-236 and -52 use hACE2 "more efficiently" than from their own species (for example, lines 48-49, 200-203, 386-388) and lines 397-398 sound like other studies that have failed to use the appropriate host species of receptor and then speculate on "alternative receptor" use (for example, PMID: 36477529 claims HKU5 may use an alternative receptor because it did not interact with a variant of ACE2 from a closely related bat species than what the virus was identified in- later shown to be false). In general, these claims should be adjusted to highlight how the appropriate ACE2 orthologue was not actually tested for -236. Maybe AlphaFold3 could be used to perform some in silico predictions.

The paragraph discussing potential alternative receptors has been deleted. We have tempered our conclusions and clarified that the appropriate ACE2 orthologue for BANAL-236 was not tested in our study (because of the unavailability of *R. marshalli* ACE2 sequence). We attempted to use AF3 to generate *in silico* predictions of the interactions between the RBDs of BANAL-236, BANAL-52, and SARS-CoV-2 and ACE2 from human and R*. malayanus*. However, AF3 was unable to generate reliable models for these interactions, as indicated by very low ipTM scores (around 0.15). For example, 3 of the 5 models proposed by AF3 for the binding interface between *R. mala*yanus ACE2 and the RBD of BANAL-52 are shown below. This result was unexpected, given that the structures of RBD/ACE2 complexes from various vertebrate species have been solved experimentally. For instance, the interactions between SARS-CoV-2 RBD and *Canis familiaris* ACE2 (PDB 7E3J), BtKY72 RBD and *R. landeri* ACE2 (PDB 8K4U), and RaTG13 RB and *Bos taurus* ACE2 (PDB 9KOW) revealed a highly conserved binding interface. It remains unclear why AF3 did not used these existing structures to predict other RBD/ACE2 interactions.

4. While R. mashalli ACE2 is not publicly available, the authors DO have a species-matched ACE2 with BANAL-52, which they could study further to strengthen their claims. For instance, I believe these experiments could be replicated in alternative cell backgrounds, such as BHK, Huh7.5, or Veros, to assess the consistency of the results. Although BANAL-52 appears to use R. malayanus ACE2 "less" than human ACE2 in figure 2E, this experiment was performed in 293T cells. For the human side of this experiment, the authors use human ACE2, human TMPRSS2 and human cells but for the bat side they are using R. malayanus ACE2, R. fer­rume­quinum TMPRSS2 and human cells. Can the authors speculate (or test) if these mixed bat factors in human cells are all properly co-localizing on the membrane as efficiently as all of the human entry factors? It may be possible that human ACE2 interacts with other human factors/microdomains involved in entry better than bat ACE2, so it seems difficult to really conclude from this one experiment that the bat virus RBD affinity for human ACE2 is really stronger.

We performed entry assays in BHK-21 cells. They were transfected with plasmids encoding the tagged versions of the entry factors and then transduced with the same S-bearing lentiviruses that were used to transduced 293T cells (novel Fig. S2E). Pilot experiments conducted with plasmids expressing GFP revealed that BHK-21 cells were far less transfected than 293T cells, with only a small percentage (around 5%) of cells being GFP positive. In contrast, nearly 100% of 293T cells were GFP positive following the same transfection protocol. This discrepancy explains why luciferase activity was approximately 2 logs lower in BHK-21 cells compared to 293T cells (novel Fig. S2E).

Despite the lower transfection efficiency, the results showed that expression of hACE2 in BHK-21 cells promoted efficient entry of lentiviruses bearing the S proteins of all 3 viruses (novel Fig. S2E), consistent with the data obtained in 293T cells (Fig. 2E). However, unlike in 293T cells (Fig. 2E), co-expression of hTMPRSS2 with hACE2 in BHK-21 cells did not enhance the entry of the lentiviruses, as compared to cells expressing hACE2 alone. Additionally, expression of rACE2 alone did not facilitate entry of lentiviruses expressing the S protein of BANAL-52 in BHK-21 cells. Given the significantly lower transfection (and possibly transduction) efficiencies in BHK-21 cells, we consider this cell lines to be less reliable for conducting entry assays. Nevertheless, despite these limitations, the use of BHK-21 cells confirmed that expression of hACE2 is required for the entry of lentiviruses expressing the S proteins of the 3 sarbecoviruses.

We performed immunofluorescence experiments in 293T cells transfected with tagged versions of human or *Rhinolophus* entry factors (hACE2-V5 and myc-hTMPRSS2, or rACE2-V5 and myc-rTMPRSS2). The cells were fixed and stained with anti-ACE2 and anti-myc antibodies. The entry factors were well expressed (novel Fig. S1C), consistent with our western blot analysis (novel Fig. S1A-B). The 4 proteins localised to the cytoplasm. An overlap between the signals of the 2 pairs of proteins was observed, suggesting a distribution consistent with a potential interaction.

We would have like to perform cell surface staining without permeabilization. However, this was not feasible as the hTMPRSS2 antibody does not cross-react with rTMPRSS2 (novel Fig. S1B) and the myc-tag is located at the cytosolic end of TMPRSS2, requiring cell permeabilization for detection.

5. BANAL-236 was obtained from a third party for this study and further passaged in Veros but there is no mention of additional whole viral sequencing to verify a pure stock without cell culture adaptations. Because Vero adaptation is very common for coronaviruses, the original BANAL-236 study isolated the virus in Veros and sequence verified after few passages to show it did not adapt to Veros. Therefore, further passaging this virus in Vero cells, as was done in this study, warrants further sequencing.

The BANAL-236 strain, initially isolated and amplified through two passages in Vero E6 cells (C2), was subjected to an additional passage in Vero E6 cells (C3) to generate a viral stock. The virus was then deep-sequenced following this third passage (C3). Only one mutation with an allelic frequency exceeding 5% was identified in the S protein: V391I (Table. S7). This mutation, which is not located in the RBD, was first detected after the second passage in Vero E6 cells at a frequency of 14.28% and subsequently became predominant by the 3^rd^ passage (87.91%). These findings have been included in the MM section.

| **Gene** | **Syn./**  **Non syn.** | **Mutation (nt)** | **Mutation (aa)** | **BANAL-236**  **C2** | **BANAL-236**  **C3** |
| --- | --- | --- | --- | --- | --- |
| ORF1a | S | G-3934-T | - | 0.00% | 1.30% |
| S | NS | G-22745-A | V-391-I | 14.28% | 87.91% |
| ORF3a | NS | A-25367-G | T-9-A | 0.00% | 3.94% |
| M | NS | C-26495-T | T-8-I | 0.00% | 1.37% |

**Novel table S7.** Frequency of allelic mutations observed in BANAL-236 C2 and C3 passages performed in VeroE6 cells. Mutations are compared to the original BANAL-236 sequence (GenBank accession number MZ937003.2) obtained from bat rectal swab. Synonymous (S) and non-synonymous (NS) mutations, and only frequencies above 1% are reported.

6. Figure 6A: The RFe-ATC cells appear to be more interferon deficient than the parental cells. Is it possible the authors have selected a slightly less immunocompetent cell line, accounting for the differences in replication phenotype in figure 3?

To investigate the potential differences in IFN response between RFe-ATC and RFe-AT cells, we conducted a transcriptomic analysis of both cell lines following stimulation with polyIC, as compared to unstimulated cells. The analysis identified 181 differentially expressed genes (DEGs, fold change ≥1.5 log2, p-value < 0.05) in stimulated RFe-AT cells compared to unstimulated control cells and 166 DEGs in stimulated ATC cells (Fig. S5A and table S1). A substantial proportion of the upregulated genes were shared between both cell lines, including ISGs that are conserved among mammalian species (such as *BST2*, *MX1*, and *IRF*s). Notably, *OAS1* and *ISG20* were among the most highly upregulated genes (Fig. 6C-D and Table S1). To identify pathways affected by poly(I:C) treatment, we performed Gene Ontology term analysis on the upregulated genes. As expected, both cell lines exhibited significant enrichment in pathways related to viral infection and IFN responses (Fig. 6E-F).

In parallel, we infected both RFe-ATC and RFe-AT cells with Japanese Encephalitis Virus (JEV), an orthoflavivirus known to circulate in multiple bat species, including *Rhinolophus* species (doi:10.3389/fcimb.2022.960507). Cells were infected with JEV at an MOI of 10, and flow cytometric analysis revealed that approximately 30% of cells were positive for the viral E protein at 24 hpi (novel Fig. S5B). RT-qPCR analysis further demonstrated a modest yet reproducible increase in the mRNA levels of *OAS1* and *ISG20* following JEV infection in both cell lines (novel Fig. S5C-D). This modest increase, compared to that observed in polyI:C-treated cells (Fig. 6A-B), is likely due to the ability of JEV to counteract the IFN response (doi:10.1128/JVI.00039-17; doi:10.1128/jvi.00077-25).

Together, these results provide evidence that both cell lines exhibited similar immunocompetence following a viral infection or polyI:C treatment. Thus, the absence of IFN induction during BANAL-236 replication is not due to a dysregulation of the IFN response in RFE-ATC cells, but rather reflects the virus’s ability to actively counteract this response.

7. I really liked the last experiment in figure 7 with the different viral genes. Nsp1, Nsp13 and Orf6 could be further tested in the RFe cells to see if they modulate the responses measured in figure 6.

We were first hoping to express BANAL-236 proteins in transfected RFe cells to study their potential ability to modulate the IFN response. Pilot experiments performed with plasmids encoding for GFP and several transfection reagents showed that the highest percentage of GFP-positive cells achieved was only 5% (see some examples below), which is insufficient to reliably assess the impact of Nsp1, Nsp13, and Orf6 on IFN responses in these cells.

We then explore several strategies to generate RFe cells stably expressing NSP1, NSP13 and Orf6. First, we cloned 4 BANAL-236 ORFs with a STREP-tag into an inducible lentiviral vector carrying an EF1α promoter: strep-NSP1, strep-NSP13, strep-ORF6, and strep-NSP8 (the latter serving as a negative control). Following transduction of RFe cells, western blot analysis revealed low expression levels of the viral proteins. These experiments, including the production of the stable RFe cells, were conducted in parallel in our lab and in Nevan Krogan’ lab. A faint signal was detected for Strep-ORF6, and an even fainter signal for strep-NSP1 and strep-NSP8 (red star below). The intensities of the marker signals between the 2 membranes allow for a comparison of the expression levels between the viral proteins and GAPDH. Strep-NSP13 was not detected in this set of experiments (rebuttal Fig. 3).

We then fused NSP1, NSP13, and ORF6 coding sequences to a FLAG-tag and inserted them into a lentiviral vector carrying an UBC promoter. A lentivirus expressing GFP was also produced to generate negative control RFe cells. Western blots analysis of RFe cells transduced with these second-generation lentiviruses revealed an improvement in NSP13 expression (novel Fig 7D). However, ORF6 expression remained low (novel Fig 7D), and most cells expressing NSP1 died, which is consistent with its known ability to block translation (doi:10.1038/s41579-022-00839-1).

To test whether Nsp13 and ORF6 could modulate IFN response in RFe cells, cells stably expressing FLAG-NSP13, FLAG-ORF6, or GFP were stimulated with polyI:C or left untreated for 16 hours. RT-qPCR analysis showed that the relative amounts of *ISG20* and *OAS1* mRNAs were significantly lower in RFe cells expressing FLAG-NSP13, compared to control cells transduced with empty lentiviruses (mock) or lentiviruses expressing GFP (novel Fig. 7E-F). These results, combined with the ones obtained with 293T cells expressing the BANAL-236 proteins (Fig. 7B), suggest that NSP13 inhibits the IFN response in a human and *Rhinolophus* cells. The inability of ORF6 to modulate ISG expression in RFe cells is likely due to its low level of expression.

**Reviewer #2**:

1.) Rigor of the BANAL-236 virus model. Interestingly, BANAL-236 is reported to use human ACE2 for cellular entry with higher efficiency compared to the ACE2 receptor from its cognate host. To strengthen this claim, it would be helpful if the authors could provide more details about how viral stocks were prepared. How many times was the virus passaged to generate working stocks used in these studies? Was the stock deep sequenced to rule out cell culture adaptive mutations (i.e., mutations that enhance affinity for African green monkey ACE2 since the virus was grown in VeroE6 cells)? There is currently no mention of these methods within the manuscript.

The BANAL-236 strain, initially isolated and amplified through two passages in Vero E6 cells (C2), was subjected to an additional passage in Vero E6 cells (C3) to generate a viral stock. The virus was then deep-sequenced following this third passage (C3). Only one mutation with an allelic frequency exceeding 5% was identified in the S protein: V391I (Table. S7). This mutation, which is not located in the RBD, was first detected after the second passage in Vero E6 cells at a frequency of 14.28% and subsequently became predominant by the 3^rd^ passage (87.91%). These findings have been included in the MM section.

| **Gene** | **Syn./**  **Non syn.** | **Mutation (nt)** | **Mutation (aa)** | **BANAL-236**  **C2** | **BANAL-236**  **C3** |
| --- | --- | --- | --- | --- | --- |
| ORF1a | S | G-3934-T | - | 0.00% | 1.30% |
| S | NS | G-22745-A | V-391-I | 14.28% | 87.91% |
| ORF3a | NS | A-25367-G | T-9-A | 0.00% | 3.94% |
| M | NS | C-26495-T | T-8-I | 0.00% | 1.37% |

**Novel table S7.** Frequency of allelic mutations observed in BANAL-236 C2 and C3 passages performed in VeroE6 cells. Mutations are compared to the original BANAL-236 sequence (GenBank accession number MZ937003.2) obtained from bat rectal swab. Synonymous (S) and non-synonymous (NS) mutations, and only frequencies above 1% are reported.

2.) Utility of the Rhinolophus fibroblast cellular model. The authors developed and characterized a novel Rhinolophus bat lung fibroblast cell model. While this presents a valuable step toward expanding bat-derived experimental systems (which are currently lacking in the field), the demonstrated utility of the model is limited. Only a single clonally derived cell line expressing human ACE2 and TMPRSS2 (RFe-ATC) were found to be susceptible to BANAL-236, and this clone was artificially engineered to express human ACE2 and TMPRSS2. Because BANAL-236 susceptibility depends on exogenous expression of human, rather than an endogenous Rhinolophus factor(s), the relevance is unclear and overall enthusiasm for this cellular model is diminished.

We have emphasized the limits of the model several times in the discussion.

3.) Mechanistic depth describing innate immune evasion pathways. Antagonism of host innate immune responses is well established for human and zoonotic coronaviruses, and these molecular mechanisms are well described. The reviewer appreciates the tremendous effort and experimental rigor in defining the innate immune antagonistic ability of BANAL-236 and the subsequent identification of the viral proteins involved in this process. However, considering the level of mechanistic detail already established for how human and zoonotic CoVs antagonize host innate immune pathways, the current study does not substantively contribute to our overall knowledge of these pathways. Additionally, only three interferon stimulated genes (OAS1, ISG20, and IFITM3) are considered in the evaluation BANAL-236’s immune evasion. **Further transcriptomic characterization is needed to define the innate immune response the RFeATC cell line**. Furthermore, experiments examining the role of specific viral proteins in innate immune antagonism are unfortunately **only conducted in HEK293T cell**s due to technical considerations. This again limits the relevance of the RFe-ATC model and weakens claims regarding the ability of BANAL-236 to evade bat innate immune responses.

To define the IFN response in RFe-ATC, and as a comparison, in RFe-AT cells, To investigate the potential differences in IFN response between RFe-ATC and RFe-AT cells, we conducted a transcriptomic analysis of both cell lines following stimulation with polyIC, as compared to unstimulated cells. The analysis identified 181 differentially expressed genes (DEGs, fold change ≥1.5 log2, p-value < 0.05) in stimulated RFe-AT cells compared to unstimulated control cells and 166 DEGs in stimulated ATC cells (Fig. S5A and table S1). A substantial proportion of the upregulated genes were shared between both cell lines, including ISGs that are conserved among mammalian species (such as *BST2*, *MX1*, and *IRF*s). Notably, *OAS1* and *ISG20* were among the most highly upregulated genes (Fig. 6C-D and Table S1). To identify pathways affected by poly(I:C) treatment, we performed Gene Ontology term analysis on the upregulated genes. As expected, both cell lines exhibited significant enrichment in pathways related to viral infection and IFN responses (Fig. 6E-F).

In parallel, we infected both RFe-ATC and RFe-AT cells with Japanese Encephalitis Virus (JEV), an orthoflavivirus known to circulate in multiple bat species, including *Rhinolophus* species (doi:10.3389/fcimb.2022.960507). Cells were infected with JEV at an MOI of 10, and flow cytometric analysis revealed that approximately 30% of cells were positive for the viral E protein at 24 hpi (novel Fig. S5B). RT-qPCR analysis further demonstrated a modest yet reproducible increase in the mRNA levels of *OAS1* and *ISG20* following JEV infection in both cell lines (novel Fig. S5C-D). This modest increase, compared to that observed in polyI:C-treated cells (Fig. 6A-B), is likely due to the ability of JEV to counteract the IFN response (doi:10.1128/JVI.00039-17; doi:10.1128/jvi.00077-25).

Together, these results provide evidence that both cell lines exhibited similar immunocompetence following a viral infection or polyI:C treatment. Thus, the absence of IFN induction during BANAL-236 replication is not due to a dysregulation of the IFN response in RFE-ATC cells, but rather reflects the virus’s ability to actively counteract this response.

We attempted to conduct experiments examining the role of specific BANAL-236 proteins in innate immune antagonism in RFe cells. Initially, we attempted to transfect RFe cells with plasmid encoding for GFP using several transfection reagents (see some examples below). The highest percentage of GFP-positive cells achieved was only 5%, which is insufficient to reliably assess the impact of Nsp1, Nsp13, and Orf6 on IFN responses in these cells.

We then explore several strategies to generate RFe cells stably expressing NSP1, NSP13 and ORF6. First, we cloned 4 BANAL-236 ORFs with a strep-tag into an inducible lentiviral vector carrying an EF1α promoter: strep-NSP1, strep-NSP13, strep-ORF6, and strep-NSP8 (the latter serving as a negative control). Following transduction of RFe cells, western blot analysis revealed low expression levels of the viral proteins. These experiments, including the production of the stable RFe cells, were conducted in parallel in our lab and in Nevan Krogan’ lab. A faint signal was detected for strep-ORF6, and even fainter signals were observed for strep-NSP1 and strep-NSP8 (red star below). The intensities of the marker signals between the 2 membranes allow for a comparison of the expression levels between the viral proteins and GAPDH. Strep-NSP13 was not detected in this set of experiments (rebuttal Fig. 3).

We then fused NSP1, NSP13, and ORF6 coding sequences to a FLAG-tag and inserted them into a lentiviral vector carrying an UBC promoter. A lentivirus expressing GFP was also produced to generate negative control RFe cells. Western blots analysis of RFe cells transduced with these second-generation lentiviruses revealed an improvement in NSP13 expression (novel Fig 7D). However, ORF6 expression remained low (novel Fig 7D), and most cells expressing NSP1 died, which is consistent with its known ability to block translation (doi:10.1038/s41579-022-00839-1).

To test whether Nsp13 and ORF6 could modulate IFN response in RFe cells, cells stably expressing FLAG-NSP13, FLAG-ORF6, or GFP were stimulated with polyI:C or left untreated for 16 hours. RT-qPCR analysis showed that the relative amounts of *ISG20* and *OAS1* mRNAs were significantly lower in RFe cells expressing FLAG-NSP13, compared to control cells transduced with empty lentiviruses (mock) or lentiviruses expressing GFP (novel Fig. 7E-F). These results, combined with the ones obtained with 293T cells expressing the BANAL-236 proteins (Fig. 7B), suggest that NSP13 inhibits the IFN response in a human and *Rhinolophus* cells. The inability of ORF6 to modulate ISG expression in RFe cells is likely due to its low level of expression.

**Part III – Minor Issues: Editorial and Data Presentation Modifications**
Please use this section for editorial suggestions as well as relatively minor modifications of existing data that would enhance clarity.

**Reviewer #1**: MINOR
1. Cell surface expression of bat ACE2 is measured by flow cytometry using human ACE2 antibodies. It might also be nice to show overall cellular protein levels by westernblot. Tagged receptors could help reduce non-specific effects from differences in ACE2 antibody cross-reactivity.

In the initial version of the manuscript, we presented western blot analysis showing the overall expression levels of hACE, rACE2, hTMPRSS2 and rTMPRSS2 in 293T cells (former Fig. S1C). These analyses revealed that the antibodies against hACE2 recognized rACE2 less efficiently than hACE2, and that antibodies against hTMPRSS2 failed to detect its *Rhinolophus* ortholog (former Fig. S1C). Consequently, we decided to tag the entry factors with V5 and myc epitopes to ensure reliable detection. We have now completed the Western blot analysis using antibodies against hACE2, hTMPRSS2, V5, and myc (novel panels S1A and S1B) to show overall expression levels of the 4 entry factors in transfected 293T cells.

Additionally, we have included Western blot analysis of RFe-AT and RFe-ATC cells, both of which express untagged versions of hACE2 and hTMPRSS2 (novel Fig. S3F).

2. Related to minor point 1, the quantitative PCR does not seem to use primers specific for R. fer­rume­quinum genes. How well do the human primers work with the bat genes?

The primers used to amplify *R. fer­rume­quinum* genes (*GAPDH, ACE2, TMPRSS2, OAS1*, and *ISG20*) are specific, as they were designed based on the *R. fer­rume­quinum* genome, as indicated in the table S6.

3. Related to major points 3 and 4, were the bat virus genes codon optimized for human cells?

No, they were not. Original sequences were used since we were planning to generate both human and bat cells expressing bat entry factors.

4. Lentiviruses can fail to faithfully recapitulate bat sarebocovirus cell entry (PMID: 26552008, supp. figure 1D). Using an alternative system like VSV may provide additional insights.

This is now discussed and the reference was added in the discussion (lines 484-86).

*‘Of note, lentivirus-based pseudotyping may not fully recapitulate the cell entry dynamics of bat sarbecovirus [58]. Therefore, using an alternative VSV-based system could provide additional insights into viral entry mechanisms.’*

5. The authors have issues maintaining expression in the bat cells and could not make a line expressing both bat ACE2 and bat TMPRSS2. What lentivector is being used? It seems like multiple promoters are being used instead of using IRES or 2A sites, which is likely driving at least some of these challenges.

We employed multiple strategies to achieve co-expression of the entry factor in RFe cells. Initially, we cloned the entry factors into lentiviral vectors containing an UBC promoter and carrying either a puromycin (ACE2) or a blasticidin (TMPRSS2) selection cassette. We tried both simultaneous and sequential transduction with the 2 lentiviral stocks. We also attempted to clone both genes into a bicistronic lentiviral vector (pLenti-UBC-ACE2-IRES-TMPRSS2). Additionally, we tested a lentiviral vector developed by the lab of Sonia Best (pWPI-IRES-Puro-Ak-ACE2-TMPRSS2, Addgene plasmid # 154987). Unfortunately, none of these strategies were successful.

6. Figure 6A and B: the colored dots are not easy to see against the gray bars. The bars could also be colored similarly.

Gray bars were replaced by coloured bars.

7. The title for supplemental figure 3 appears to have a find and replace error from adjusting the name of the cell line: "InteRFeron"

This was corrected.

8. VSV could be an interesting virus to use in the bat cells to show interferon response to infection. It is fairly immuno-stimulatory in many cell lines.

We have infected RFe, RFe-AT, and RFe-ATC with VSV and performed RT-qPCR analysis to assess *ISG20* and *OAS1* mRNA levels. Unexpectedly, no induction of *ISG20* nor *OAS1* was detected, despite active VSV replication in these 3 cell lines (see Fig. 3G). Additionally, no ISG upregulation was observed in Caco-2 cells infected with the same VSV stock. For this reason, we performed experiments using JEV instead (see above novel Fig. 5B-D).

**Reviewer #2**: 1.) The author’s report that RFe-ATC cells are difficult to transfect. Have they tried additional transfection reagents shown to be effective for otherwise hard-to-transfect cell lines? If so, please mention.

We indeed tried several transfection reagents, including some designed for hard-to-transfect cells (GeneJammer), as shown above (rebuttal Fig. 2). This is now mentioned in the text (lines 521-524).

*‘Assessing the IFN-evasion capacity of these proteins in RFe cells proved challenging due to their low transfection efficiency with multiple reagents (Lipofectamine, Lipojet, Jetprime and Genejammer).’*

2.) The use of both full-length virus and pseudotyped viruses strengthens the rigor of the work. However, the figure legends are not immediately apparent to the reader which platform was used in each experiment.

We have modified the figure legends to specify when lentiviruses were used in the experiments (Fig. 2 and Fig. S2E).
